# Editorial: Brain and Somatization Symptoms in Psychiatric Disorders

**DOI:** 10.3389/fpsyt.2019.00146

**Published:** 2019-03-28

**Authors:** Xiaoya Fu, Fengyu Zhang, Feng Liu, Chaogan Yan, Wenbin Guo

**Affiliations:** ^1^Department of Psychiatry, The Second Xiangya Hospital of Central South University, Changsha, China; ^2^The Global Clinical and Translational Research Institute, Bethesda, MD, United States; ^3^Peking University Huilongguan Clinical Medical School and Beijing Huilongguan Hospital, Beijing, China; ^4^Department of Radiology, Tianjin Medical University General Hospital, Tianjin, China; ^5^Magnetic Resonance Imaging Research Center, Institute of Psychology, Chinese Academy of Sciences, Beijing, China

**Keywords:** structural MRI, functional MRI, somatization symptoms, depression, anxiety

Somatization is the expression of mental phenomena as physical (somatic) symptoms characterized by “physio-somatic” symptoms that result in significant distress and/or problem functioning [DSM V, (1)]. These physical symptoms, which may not be associated with a diagnosed medical condition, are medically unexplained (2). Somatization is very common in psychiatric disorders including depression (3), anxiety, and panic disorder and associated with functional impairment, increased disability and high health care cost (2, 4, 5). Because individuals with somatization tend to seek medical help, it poses a significant medical, social, and economic burden (6).

Somatization is strongly associated with depressive and anxiety symptoms, intermediately associated with symptoms of schizophrenia and mania, and has the weakest association with symptoms of substance use and antisocial personality (7). In primary care, depression is the most common comorbid disorder associated with somatization (8). One large-scale study of primary care patients showed that 69% of depressed individuals have somatic symptoms, whereas an increased number of somatization symptoms is associated with a higher risk of depression (9, 10). Although many psychiatric illnesses, such as depression and schizophrenia, were considered biologically-mediated disorders, somatization was still regarded as functional distress without a biological substrate. Previous studies have observed some structural change and functional disturbance of the brain in patients with somatization (11–13), but the neuropathology underlying somatization symptoms in psychiatric disorders remains unclear.

In recent decades, a growing body of work has demonstrated regional and illness-specific brain changes at the onset of psychiatric disorders and in individuals at risk for such disorders. Studies of the change in the brain with a focus on differential diagnosis, illness severity, and treatment outcome in psychiatric disorders may be of considerable clinical significance. Cerebral deficits mainly relate to brain function as well as gross anatomic alterations, but the changes may be modest, requiring quantitative analysis instead of just routine visual inspection of images. Therefore, it is urgent to develop and use noninvasive quantitative means to observe patterns of functional and structural cerebral changes in psychiatric patients. These advances help understand the neuropathy of psychiatric disorders further and provide a direction for the development of objective quantitative measures of patterns of brain abnormalities in psychiatric disorders.

In this special issue of “Brain and Somatization Symptoms in Psychiatric Disorders,” 27 articles are published (Table 1). There are 25 research articles, one systematic review, and one case report. Except for one study on animals, all are studies in humans; and they are primarily comprised of neuroimaging study of patients with somatization disorders, major depressive disorder (MDD), substance use, insomnia and pain, schizophrenia and cognitive function. One study was designed with the interventional component; the others are cross-sectional studies of patients with matched controls for comparisons. The review paper was on suicidal behaviors.

**Table 1:** Characteristics of the publications.

**Category of diseases**	**Disease**	**Design**	**No Articles**
Somatization disorder or somatic disorder	Somatization disorder Multiple myelomas Ashmatic disorder, Renal disease Systematic lupus erythematosus (SLE)	Case-control Case-report	5
Depressive disorder	Major depressive disorder Bipolar depressive disorder Unipolar depressive disorder Menoposal depressive rate	MECT^*^ Case-control	7
Substance use	Abstinence of Methamphetamine Methamphetamine Users Ketamine-dependent subjects Smokers	Case-control	5
Insomnia and pain	Insomnia Insomnia with pain Insomnia with depression	Case-control	4
Schizophrenia		Case-control	2
Other disorders	Bipolar disorder	Case-control	1
	Post-traumatic stress disorder	Case-control	1
	Mild cognitive impairment	New methods	1
	Suicidal behavior	Systematic review	1

* *Intervention with MECT, modified electroconvulsive therapy treatment on patients*.

## Somatization and Somatic Disorders

Several articles are on imaging studies of somatization disorder (SD) or somatic diseases (e.g., systematic lupus erythematous, asthmatic disorder, renal disease, and multiple myeloma), but only one directly focuses on SD. Suspecting that patients with SD may have anatomical deficits in the cortico-limbic-cerebellar circuit, which may affect the connectivity of the circuit, Li et al. report structural alteration, which partially affects the connectivity of the cortico-limbic-cerebellar circuit, in first-episode and drug-naive patients with SD compared with healthy controls. The structural alteration and deficits in connectivity are correlated with cognitive performance as measured by the Wisconsin Card Sorting Test (WCST) in the patients with SD, although the evidence was suggestive in terms of p-value and a sample size of 26 patients.

Three studies show structural and neuropathological alterations in somatic disorders. Using resting-state functional magnetic resonance imaging (rs-fMRI), Liu et al. examine the change in brain activity in patients with systemic lupus erythematosus (SLE) but without major neuropsychiatric manifestations (non-NPSLE patients, *n* = 118), and healthy controls (HC, *n* = 81). As an autoimmune disease, individuals with SLE may experience a variety of somatic symptoms that include fatigue, pain or swelling in the joints, and other symptoms such as sun sensitivity, seizures, and psychosis. Based on the measure of regional homogeneity (ReHo), decreased ReHo is found in the fusiform gyrus and thalamus but an increased ReHo is in the parahippocampal gyrus and uncus, and the SLE disease activity index was positively correlated with the ReHo measure in the cerebellum and negatively correlated with that in the frontal gyrus. Several brain areas showed correlations with depressive and anxiety statuses. Additionally, Yin et al. report widespread impairment of the white matter in end-stage renal disease patients, which may result from the accumulation of serum creatinine and blood urea nitrogen; and damage to the thalamic radiation, corona radiata and the reduced integrity of left anterior thalamic radiation may affect cognitive function, working memory and executive function. Moreover, Zhang et al. compare cerebral blood flow between depressed asthmatic and non-depressed asthmatic patients and find that depressed asthmatic patients show an increase in regional cerebral blood flow in the right cerebellum posterior lobe.

In addition, Yao et al. report the case of a 57-year-old woman with pain and discomfort in multiple sites of the upper body, which had been misdiagnosed as somatic symptom disorder for 6 months. After imaging examinations of all painful parts, she was eventually diagnosed with multiple myeloma. This highlights the importance of completing imageological examinations of all the painful parts, especially when symptoms are associated with objective signs and treatment has been ineffective.

## Depressive Disorders

Two articles focus on studies of negative interpretation and depressive symptoms, regional abnormality in major depressive disorder. Zhou et al. show an association between interpretative biases and depressive symptoms in older adults by using the method of event-related brain potentials (ERPs). According to cognitive theories, the tendency that depressed populations have negative interpretations on ambiguous stimuli, situations, and events may play an important role in both development and maintenance of depression. Zuo et al. report that the medial orbitofrontal cortex and posterior cingulate cortex were thicker in patients with repeated-episode MDD than those with first episode MDD. They also find regional abnormalities of the frontal-limbic circuits in patients with MDD compared with healthy controls.

In addition, Li et al. find that the global functional connectivity density (gFCD) significantly increased in the posterior-middle insula, the supramarginal gyrus, and the dorsal medial prefrontal cortex (dmPFC) in patients with MDD compared to healthy controls. However, gFCD statistically increased in the perigenual anterior cingulate cortex (pgACC), the orbitofrontal cortex bilaterally and the left-supramarginal gyrus but decreased in the posterior insula after modified electroconvulsive therapy (MECT). The gFCD in the pgACC and the right orbital frontal cortex of the depressive group before MECT was positively associated with HAMD scores after MECT. Wu et al. find that abnormal myelin oligodendrocyte glycoprotein (MOG) might be an important factor in white matter damage in patients with different onset age of drug-naive depression.

There are two papers focusing on an imaging study of bipolar and unipolar depressive disorders. Fu et al. examine hemodynamic changes between the task and rest period in patients with bipolar depression during the Tower of London task and the verbal fluency task using near-infrared spectroscopy. The Tower of London task is one of the most commonly used tests for evaluating executive functions and can indicate planning and problem-solving abilities. The results indicate that planning and problem-solving dysfunction is related to the impairment of the prefrontal cortex in patients with bipolar depression. Qiu et al. evaluate the fractional amplitude of low-frequency fluctuations based on resting-state functional magnetic resonance in patients with bipolar depression and unipolar depressive disorder. In addition, Gu et al. conducted an animal study and investigated the interactions between neuromodulators and sex hormone involved in menopause-related depression in rats.

## Substance Use Disorder

Of five papers that focus on substance use disorders, two studies investigate the structural alteration in brains of patients with long-term abstinence of methamphetamine (MA) use disorder and healthy controls. The effects of long-term abstinence of MA structures are described by Zhang et al., and they find changes in gray matter volume from visual and cognitive function regions. This article also reports a positive correlation between gray matter volume of the left cerebellum crus and the duration of abstinence, suggesting that prolonged abstinence is beneficial to cognitive function recovery. Huang et al. find that individuals with MA use disorder following long-term drug rehabilitation have increased activation in the occipital lobe when exposed to pornographic cues compared to MA cues, which illustrate that the libido brain response might be restored, and that sexual demand might be more robust than drug demand.

In addition, two additional studies examine the metabolites in human brains of MA users and healthy controls. Wu et al. examine the metabolites alterations in the medial prefrontal cortex of MA users and find that the MA group shows a significant reduction in the ratio of n-acetyl-aspartate (NAA)/phosphocreatine plus creatine (PCr+Cr), but an elevation in the ratios of glutamate (Glu)/PCr+Cr and myo-inositol (mI)/PCr+Cr ratio, compared with healthy controls, suggesting that Glu may play a key role in methamphetamine-induced neurotoxicity. Yang et al. investigate absolute glutamate concentrations and metabolite ratios in patients with MA addiction in comparison with healthy controls. They find that the ratio of glutamate-to-creatine in the brainstem is significantly elevated in the MA group, and glutamate concentrations in the brainstem are also significantly elevated and are positively correlated with the duration and total dose of regular addiction in MA group.

Moreover, one study focuses on chronic ketamine-dependent subjects and cigarette smokers. Liao et al. examine the effects of cue exposure on different drugs to identify the reliable patterns of activation in a particular sample or specific drug-related cue exposure paradigm and report that ketamine users and smokers show significantly increased activation in the anterior cingulate cortex in response to ketamine cues but lower activation in response to sexual cues, which may partly reflect the neural basis of sexual dysfunction.

## Insomnia and Pain

Insomnia is often observed to accompany somatic complaints including pain. Four papers published in this special issue are on insomnia. Wei et al. investigate the effect of a bad night's sleep on pain increases alongside insomnia severity. Using data from 3,508 volunteers (2,684 females, mean age of 50 years), they show that people suffering from more severe habitual insomnia have stronger mutual within-day reactivity of pain than did poor sleepers. Using graph-based approaches, Ma et al. investigate topological abnormalities of functional brain networks in individuals with primary insomnia (PI) and healthy controls. Ma et al. show PI is associated with the abnormal organization of large-scale functional brain networks, which may account for memory and emotional dysfunction in people with primary insomnia. Liu et al. attempt to explore the neural mechanisms underlying the multifaceted interplay between insomnia and depression based on major depressive patients with high or low insomnia and healthy controls, and suggest that increased resting state increased amplitude of low-frequency fluctuation in the right inferior frontal gyrus/anterior insula may be related explicitly to the hyperarousal state of insomnia in patients with MDD, independently of the effects of anxiety and depression. EEG microstate assessment could provide objective markers of subjective experience dimensions in studies on consciousness during the transition between wake and sleep when self-reporting is not possible because it would interfere with the very process under study. Wei et al. also show the associations of electroencephalography microstate properties with somatic awareness and increased somatic awareness in insomnia.

## Schizophrenia

Two studies are performed on imaging and imaging genetic study of schizophrenia. Using the regional homogeneity approach, Gao et al. conducted a study of resting-state functional magnetic resonance imaging (RS-fMRI) to examine differences in neural activity of brain regions between patients with treatment-resistant schizophrenia (TRS) and non-treatment-resistant schizophrenia (NTRS), and found widespread differences in ReHo among the three groups of TRS, NTRS, and healthy controls in the occipital, frontal, temporal, and parietal lobes.

Regional spontaneous neuronal activity, measured as regional homogeneity (ReHo), has been consistently reported in patients with schizophrenia (SCZ) and their unaffected siblings. Gou et al. found significant interactions between the genotype of SNPs at the gene *DISC1* and schizophrenia case-control status in three of six SNPs on regional homogeneity in a combined schizophrenia and control sample in multiple regions of the brain. For example, rs821617 shows a significant interaction on ReHo in the right precuneus (PCUN), middle occipital gyrus (MOG), basal ganglia (BG), post-central gyrus (PostCG), left precentral gyrus (PreCG), and calcarine (CAL). Of G allele carriers, SCZ patients are shown to have lower ReHo in the right PCUN, MOG, and left CAL compared to HC, whereas no significant difference in ReHo was observed between SCZ and HC group in A-allele homozygous. The G-allele carriers are also shown to have lower ReHo in most of the regions mentioned above, whereas the HC group showed the opposite findings in some of those regions.

## Other Disorders and Conditions

Four studies focus on other disorders and conditions including bipolar II disorder, post-traumatic stress disorder (PTSD), suicidal behaviors (SB), and mild cognitive impairment (MCI). Luo et al. examine the functional connectivity between the cerebellum and cerebrum, particularly the central executive network (CEN) and the default-mode network (DMN) in patients with unmedicated bipolar II disorder. They then show disrupted functional connectivity between the cerebellum and the CEN (mainly in the left dorsal lateral prefrontal cortex and anterior cingulate cortex) and DMN (mainly in the left medial prefrontal cortex and temporal lobe), suggesting the significant role of the cerebellum-CEN and -DMN connectivity in the pathogenesis of bipolar II disorder. Assuming that the disordered communication between vulnerable brain regions or networks might contribute to the abnormalities in PTSD patients, Weng et al. explore the temporal propagation patterns of brain activity in patients with PTSD with the methods of resting-state lag analysis and resting-state functional MRI analysis for comparison and supplementing each. A systematic literature search across four databases was performed to identify all English-language neuroimaging articles involving patients with at least one psychiatric diagnosis and assessment of suicidal behaviors (SB) or non-suicidal self-injury (NSSI), Domínguez-Baleón et al. show that suicidality is associated with the frontal and temporal cortex in 15 (45%) and 9 (27%) of 33 studies across four disorders including MDD, schizophrenia, bipolar disorder, and borderline personality disorder, but no single studies focus on NSSI. In addition, Bi et al. propose a novel method of the weighted random support vector machine cluster, in which multiple support vector machines were built and different weights were given to corresponding support vector machines with different classification performances. They evaluated their algorithm on resting-state functional magnetic resonance imaging data of 93 mild cognitive impairment patients.

In summary, we obtained good coverage of the neuroimaging study of adult-onset psychiatric disorders but had relatively less of a focus on somatization. In addition, despite this, most of these studies used healthy controls, and few employed interventional approaches to study the effect before and after the intervention.

## Author Contributions

All authors listed have made a substantial, direct and intellectual contribution to the work, and approved it for publication.

### Conflict of Interest Statement

The authors declare that the research was conducted in the absence of any commercial or financial relationships that could be construed as a potential conflict of interest.
